# Isolation and characterization of cefotaxime resistant *Escherichia coli* from household floors in rural Bangladesh

**DOI:** 10.1016/j.heliyon.2024.e34367

**Published:** 2024-07-09

**Authors:** Tahani Tabassum, Md. Sakib Hossain, Ayse Ercumen, Jade Benjamin-Chung, Md. Foysal Abedin, Mahbubur Rahman, Farjana Jahan, Munima Haque, Zahid Hayat Mahmud

**Affiliations:** a Laboratory of Environmental Health, Health Systems and Population Studies Division, International Centre for Diarrhoeal Disease Research, Bangladesh (icddr,b), Dhaka, 1212, Bangladesh; b Biotechnology Program, Department of Mathematics and Natural Sciences, BRAC University, Merul Badda, Dhaka, Bangladesh; c Department of Forestry and Environmental Resources, Global Water, Sanitation and Hygiene Cluster, NC State University, Raleigh, NC, 27607, USA; d Department of Epidemiology & Population Health, Stanford University School of Medicine, CA, 94305-5101, USA; e Environmental Health and WASH, Health Systems and Population Studies Division, International Centre for Diarrhoeal Disease Research, Bangladesh (icddr,b), Dhaka, 1212, Bangladesh; f Global Health and Migration Unit, Department of Women's and Children's Health, Uppsala University, Sweden

**Keywords:** AMR, MDR, XDR, ESBL *E. coli*, Resistance genes, Virulence genes, Household floors, Biofilm

## Abstract

Antimicrobial resistance (AMR) is a rising health concern worldwide. As an indicator organism, *E. coli*, specifically extended-spectrum β-lactamase (ESBL) producing *E. coli*, can be used to detect AMR in the environment and estimate the risk of transmitting resistance among humans, animals and the environment. This study focused on detecting cefotaxime resistant *E. coli* in floor swab samples from 49 households in rural villages in Bangladesh. Following isolation of cefotaxime resistant *E. coli*, DNA extracted from isolates was subjected to molecular characterization for virulence and resistance genes, determination of resistance to multiple classes of antibiotics to define multidrug resistant (MDR) and extensively drug resistant (XDR) strains, and the biofilm forming capacity of the isolates. Among 49 households, floor swabs from 35 (71 %) households tested positive for cefotaxime resistant *E. coli*. Notably, all of the 91 representative isolates were ESBL producers, with the majority (84.6 %) containing the *bla*_*CTX-M*_ gene, followed by the *bla*_*TEM*_ and *bla*_*SHV*_ genes detected in 22.0 % and 6.6 % of the isolates, respectively. All isolates were MDR, and one isolate was XDR. In terms of pathogenic strains, 8.8 % of the isolates were diarrheagenic and 5.5 % were extraintestinal pathogenic *E. coli* (ExPEC). At 25 °C, 45 % of the isolates formed strong biofilm, whereas 43 % and 12 % formed moderate and weak biofilm, respectively. On the other hand, at 37 °C, 1.1 %, 4.4 % and 93.4 % of the isolates were strong, moderate and weak biofilm formers, respectively, and 1.1 % showed no biofilm formation. The study emphasizes the importance of screening and characterizing cefotaxime resistant *E. coli* from household floors in a developing country setting to understand AMR exposure associated with floors.

## 1 Introduction

The rising trend of antimicrobial resistance (AMR) in bacterial pathogens has become a worldwide health concern, significantly raising the mortality and morbidity rate of microbial infections [1]. Competition for resources among microorganisms and the production of secondary metabolites to enhance survival, fueled by the acquisition of resistance through horizontally transferring genes within neighboring bacterial populations, results in both established and emerging mechanisms of resistance among bacteria [2]. The resultant multidrug resistant patterns in bacteria lead to infections that are difficult to treat with existing third generation antimicrobials, posing severe global health concerns.

*Escherichia coli*, a common resident within warm-blooded animals’ gastrointestinal tract, is the most widely utilized fecal indicator organism worldwide to assess contamination in environmental compartments [3]. *E. coli* has also been classified as one of the organisms most commonly involved in horizontally transferring antibiotic resistance genes, therefore playing a significant part in the emergence and spread of AMR [4]. AMR *E. coli* can serve as a suitable indicator to detect and quantify AMR in the environment, assess how anthropogenic activities affect AMR in specific environmental compartments and estimate the risk of transmission between the environment, humans and animals/livestock [5]. Specifically, extended spectrum β-lactamase (ESBL) producing *E. coli* is employed in national and international AMR surveillance programs, as per WHO recommendations [6]. Although most *E. coli* strains coexist with their human host with mutual benefits, six pathogenic strains are often associated with food-borne infections in humans [7]. Other pathotypes of *E. coli* responsible for extraintestinal infections have been termed extraintestinal pathogenic *E. coli* (ExPEC). Studies suggest that ExPEC is the leading cause of adult bacteremia, the second leading cause of neonatal meningitis (meningitis-associated *E. coli,* MNEC) and is responsible for the vast majority of urinary tract infections (uropathogenic *E. coli*, UPEC) [8]. A study demonstrated that the severity of sepsis induced by ESBL ExPEC is three times higher than β-lactam resistant *E. coli* strains [9].

In settings where human/animal fecal waste is not isolated from the environment, antimicrobial resistant organisms can be transmitted to new hosts from feces via multiple environmental compartments [10]. Among these compartments, soil may be a particularly important reservoir. Domestic animal ownership, open defecation, poor sanitation practices and the presence of animal fecal waste have been associated with *E. coli* contamination of soil [[11], [12], [13], [14], [15]], and levels of contamination within household interiors can be extensive in developing countries [13,16,17]. In rural areas of developing countries like Bangladesh, household floors are frequently made of soil and can mediate direct contact of residents with contaminated soil. A recent study in rural Bangladesh that collected front yard soil detected multidrug resistant *E. coli* in 12.6 % of samples and potentially pathogenic *E. coli* in 10 % of samples [12]. The persistence of AMR *E. coli* in domestic environments is a threat because the organism produces filamentous structures from the cell surface that help it adhere to surfaces [5]. AMR in soil is particularly concerning because contaminated soil in turn can contaminate hands and objects in the home and soil can also be ingested deliberately (geophagia) or as dust [11,[13], [14], [15],^18^].

The WHO has long highlighted that in order to estimate AMR threat to animal and human health, a multi-sectoral “One-Health” approach to AMR surveillance is required [1]. Considering the suitability of *E. coli* to assess the burden of AMR and microbial contamination in household settings, this study focused on detecting and isolating cefotaxime resistant *E. coli* from household floors in rural Bangladesh to assess their multidrug resistance profiles, presence of pathogenic and resistance genes, correlation among phenotypic and genotypic traits, as well as their biofilm forming capacity. Cefotaxime has been proposed to be a cost-efficient alternative for detecting presumptive ESBL *E. coli* which may confer resistance to antibiotics from multiple classes [19], hence this antibiotic was utilized for assessing β-lactamase producing capability.

## 2 Results

### 2.1 Enumeration of cefotaxime resistant *E. coli* through MPN method

Out of the 49 households in our study, floor swabs from 35 (71 %) households were positive for cefotaxime resistant *E. coli* based on the detection of fluorescent cells from the Quanti-Tray/2000 system supplemented with cefotaxime solution. The counts of cefotaxime resistant *E. coli* per positive household are provided in Supplementary Table S1. The geometric mean of cefotaxime resistant isolates across the 35 positive households was >3.6 × 10^3^ MPN per 100 ml of floor swab elution. Cefotaxime resistant *E. coli* counts ranged from 10 MPN/100 mL floor swab elution (observed among five households) to above the upper detection limit of 24,196 MPN/100 mL floor swab elution (observed among three households).

### 2.2 ESBL genes were predominant among cefotaxime resistant *E. coli* isolates

The representative isolates obtained were already presumptive ESBL producers considering their resistance to cefotaxime in the modified IDEXX assay supplemented with cefotaxime. Further molecular assessment showed that a high percentage of the commonly distributed ESBL genes were present within the isolates. The distribution of the detected β-lactamase genes and the percentage of isolates harboring different combinations of ESBL genes are demonstrated in Table 1. Among the 91 isolates, 84 (92.3 %) could be classified within the three most common ESBL producing genes, including 77 (84.6 %) positive for *bla*_*CTX-M*,_ 20 (22 %) for *bla*_*TEM*_, and 6 (6.6 %) for *bla*_SHV_. Among these, 14 isolates (15.4 %) were positive for both *bla*_*CTX-M*_ and *bla*_*TEM*_, and 3 (3.3 %) for all three genes (*bla*_*CTX-M*_, *bla*_*TEM*_ and *bla*_*SHV*_). No isolates were positive for *bla*_OXA_.

**Table 1:** Distribution of β-lactamase genes among *E. coli* isolates.

ESBL genes and combinations	Percentage of isolates
CTX-M	84.6 % (77/91)
TEM	22.0 % (20/91)
SHV	6.6 % (6/91)
OXA	0.0 % (0/91)
CTX-M and TEM	15.4 % (14/91)
CTX-M and SHV	0.0 % (0/91)
CTX-M and OXA	0.0 % (0/91)
TEM and SHV	0.0 % (0/91)
TEM and OXA	0.0 % (0/91)
SHV and OXA	0.0 % (0/91)
CTX-M, TEM and SHV	3.3 % (3/91)
TEM, SHV and OXA	0.0 % (0/91)
SHV, OXA and CTX-M	0.0 % (0/91)
CTX-M, TEM, SHV and OXA	0.0 % (0/91)

### 2.3 Highly-drug resistant *E. coli* strains present in the soil swabs

**Fig. 1:**
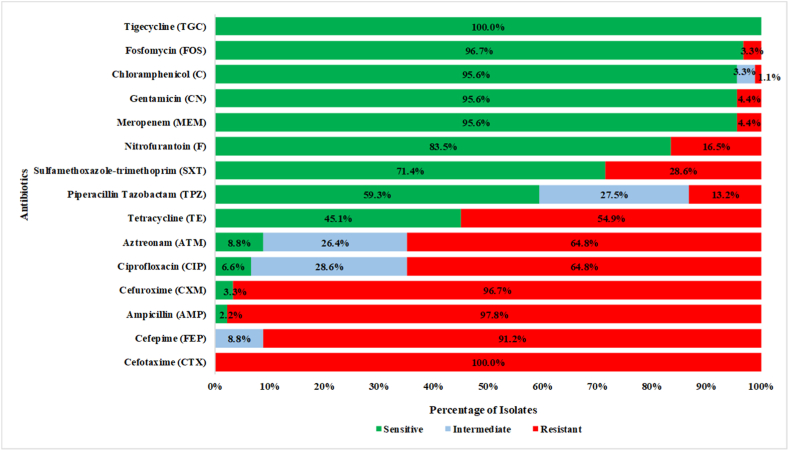
Multiple-drug resistance profile of *E. coli* isolates. Considering the selective pressure induced by cefotaxime-addition in the modified-IDEXX assay, all the isolates showed phenotypic resistance to cefotaxime. Majority of the isolates showed resistance to three other antibiotics, cefepime, ampicillin, and cefuroxime. On the other hand, a number of isolates showed sensitivity to tigecycline, fosfomycin, chloramphenicol, gentamicin, and meropenem.

The Kirby-Bauer disc diffusion assay demonstrated that the majority of the 91 isolates were resistant to multiple classes of antibiotics based on CLSI and EUCAST guidelines. Among the 15 tested antibiotics, 91/91 (100 %) of the isolates were susceptible to tigecycline and resistant to cefotaxime. For the remaining 13 antibiotics, the majority of the isolates were resistant to the following three antibiotics: 89/91 (97.8 %) to ampicillin, 88/91 (96.7 %) to cefuroxime, and 83/91 (91.2 %) to cefepime. On the other hand, the majority of the isolates were sensitive to four antibiotics: 88/91 (96.7 %) to fosfomycin, 87/91 (95.6 %) to chloramphenicol, 87/91 (95.6 %) to gentamicin, and 87/91 (95.6 %) to meropenem. All isolates were multidrug resistant (resistant to ≥ 3 antimicrobial categories) [[20], [21], [22], [23]], and one isolate was extensively drug resistant (XDR) (resistant to ≥ 12 antimicrobial classes) [24]. The percentage of resistance among the isolates is depicted in Fig. 1.

### 2.4 Diarrheagenic *E. coli* were prevalent on household floors

**Fig. 2:**
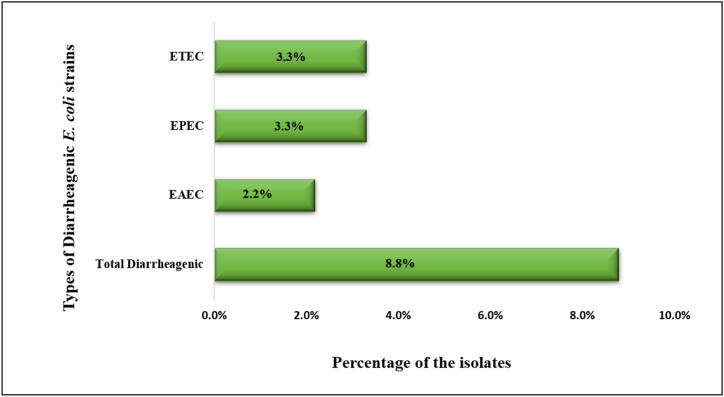
Classification of the diarrheagenic *E. coli* isolates. No EHEC and EIEC were found among the isolates. Among the persistent types of diarrheagenic isolates, ETEC and EPEC were more prevalent.

Screening of cefotaxime resistant *E. coli* isolates for intestinal pathogenic genes showed that 8/91 isolates (8.8 %) appeared to be diarrheagenic. Among those, two isolates (2.2 %) were identified as EAEC and positive for both *aaiC* and *aat* genes; three isolates (3.3 %) were identified as EPEC and positive for both *bfpA* and *eae* genes; three isolates (3.3 %) were identified as ETEC and positive for *eltB* gene. None of the isolates were positive for *eltB*, *iaa, ipaH*, *stx1,* and *stx2* genes. The percentage of diarrheagenic isolates is depicted in Fig. 2.

### 2.5 Cefotaxime resistant *E. coli* isolates with ExPEC genes

**Fig. 3:**
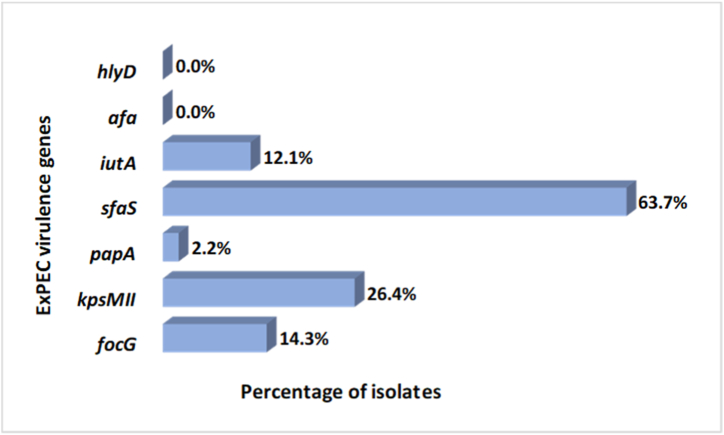
Distribution of ExPEC genes among *E. coli* isolates. Predominantly, *sfaS* gene was present among the isolates, followed by *kpsMII* gene. None of the isolates contained *hlyD* and *afa* genes.

A high prevalence of ExPEC genes was detected among the cefotaxime resistant *E. coli* isolates. Five out of the seven targeted genes were detected, including *focG, kpsMII, sfaS, papA* and *iutA* genes. The characteristics of the ExPEC genes among the isolates are depicted in Fig. 3. Out of the 91 isolates, 77 isolates (84.6 %) were positive for at least one ExPEC gene, 21 isolates (23.1 %) for at least two genes and 5 isolates (5.5 %) for at least 3 genes. Notably, 13 isolates (14.3 %) were positive for *focG*, 24 (26.4 %) were positive for *kpsMII*, 2 (2.2 %) for *papA*, 58 (63.7 %) for *sfaS*, and 11 isolates (12.1 %) were positive for *iutA*. None of the isolates were positive for *afa* and *hlyD*.

Different combinations of genes were detected amongst the isolates (Table 2). A total of 21 isolates demonstrated the presence of two different genes. Of these, 4 isolates (4.4 %) were positive for both *sfaS* and *iutA*, 4 isolates (4.4 %) were positive for both *focG* and *sfaS*, 4 isolates (4.4 %) were positive for both *iutA* and *kpsMII*, 9 isolates (9.9 %) were positive for both *kpsMII* and *sfaS.* Similarly, 2 isolates (2.2 %) were positive for *kpsMII, focG* and *sfaS,* 2 isolates (2.2 %) were positive for *kpsMII, papA* and *iutA*, and 1 isolate was positive for *focG, iutA*, and *sfaS*. If an *E. coli* isolate harbors at least three ExPEC genes, then it is classified as an extraintestinal pathogen or ExPEC strain [25]. According to these criteria, 5 isolates (5.5 %) were ExPEC strains among the 91 isolates.

**Table 2:** Combinations of ExPEC genes among the cefotaxime resistant *E. coli* isolates.

Number of ExPEC genes	Combinations of ExPEC genes	Percentage of *E. coli* isolates
Two genes	*sfaS + iutA*	4.4 % (4/91)
	*focG + sfaS*	4.4 % (4/91)
	*kpsMII + sfaS*	9.9 % (9/91)
	*kpsMII + iutA*	4.4 % (4/91)
Three genes	*kpsMII + papA + iutA*	2.2 % (2/91)
	*focG + kpsMII + sfaS*	2.2 % (2/91)
	*focG + iutA + sfaS*	1.1 % (1/91)

### 2.6 Biofilm formation ability of cefotaxime resistant *E. coli* isolates

**Fig. 4:**
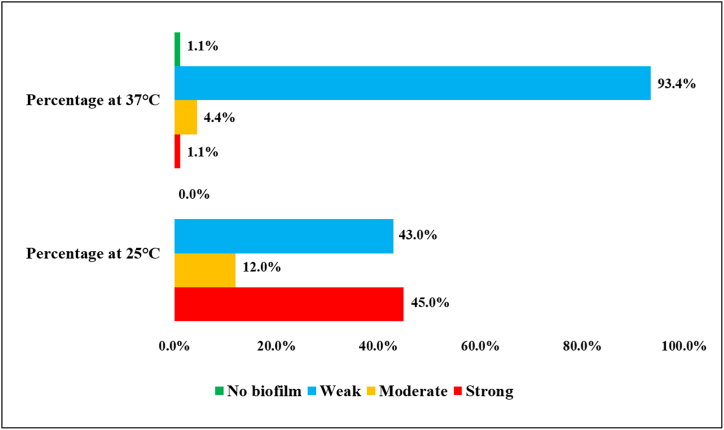
Biofilm formation of cefotaxime resistant *E. coli* isolates at 37 °C and 25 °C demonstrates biofilm forming capacity at 25 °C is comparatively higher.

Higher biofilm formation was observed at 25 °C compared to 37 °C. The percentage of isolates demonstrating variable strength of biofilm formation at different incubation temperatures is depicted in Fig. 4. At 25 °C, 41/91 (45 %) of the isolates formed strong biofilm, 39/41 (43 %) formed moderate biofilm, and 11/41 (12 %) formed weak biofilm. On the other hand, at 37 °C, a very low percentage of isolates showed a biofilm forming tendency. At this temperature, 1/91 (1.1 %) of the isolates formed strong biofilm, 4/91 (4.4 %) formed moderate biofilm, 85/91 (93.4 %) formed weak biofilm, and 1/91 (1.1 %) formed no biofilm.

### 2.7 Correlation between phenotypic and genotypic features

**Fig. 5:**
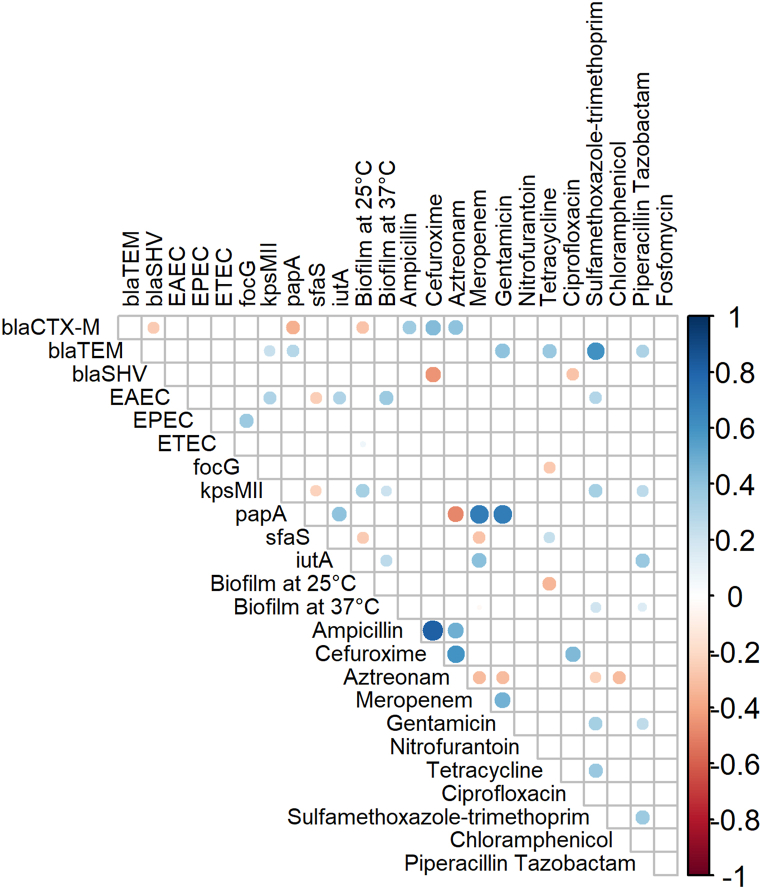
Correlation matrix of the genotypic (resistance and virulence genes) and the phenotypic (antibiotic resistance profiles and biofilm formation) features demonstrates a significant correlation among the variables. White spaces are non significantly correlated. Blue circles indicate a significant positive correlation and red circles indicate a significant negative correlation. The size and strength of color represent the numerical value of the correlation coefficient. The matrix only demonstrates significant (p < 0.05) associations, as assessed by the Fisher Exact test.

To assess the correlation between the phenotypic attributes (biofilm formation and antimicrobial resistance) and genotypic attributes (resistance and virulence genes), a correlation matrix was constructed (Fig. 5). Among the β-lactams, resistance to ampicillin, cefuroxime and aztreonam was positively correlated to the presence of *bla*_*CTX-M*_ genes but negatively correlated or non-correlated to the presence of *bla*_*SHV*_ and *bla*_*TEM*_ genes, respectively. Resistance to sulfamethoxazole-trimethoprim was significantly positively correlated to the presence of *bla*_*TEM*_ gene.

Significant correlations were also detected in resistance amongst multiple antibiotics, which complies with their phenotypic patterns of resistance. Resistance to almost all classes of antibiotics showed some positive correlation with other classes, except chloramphenicol, fosfomycin, tetracycline and nitrofurantoin. Except for one isolate, all other isolates showed similar resistance to ampicillin and cefuroxime, thereby resistance to these two antibiotics showed a significant positive correlation. The presence of the *papA* gene was strongly correlated with resistance against carbapenems and aminoglycosides. In contrast, the presence of *bla*_*SHV*_ and *papA* genes showed a negative correlation with cefuroxime and aztreonam, respectively. The coexistence of resistance and virulence was also detected within some isolates. In terms of biofilm forming ability, *kpsMII, iutA*, and EAEC strains showed a significant correlation with biofilm formation at 37 °C. At 25 °C, only *kpsMII* showed a significant positive correlation, whereas resistance to tetracycline and presence of *bla*_*CTX-M*_ and *sfas* genes showed significant negative correlations with biofilm formation.

## 3 Discussion

AMR has become a rising concern for the world of health sciences in this century and multiple studies have detected the presence of pathogenic *E. coli* strains on surfaces and soils inside rural households, as well as on hands and in stored drinking water in lower-income countries [13,16,17,26]. Antimicrobial resistant *E. coli* has been detected in courtyard soil in Bangladesh, indicating soilborne presence of AMR [12]. In our study in rural Bangladesh, out of 49 households, 35/49 (71 %) of indoor household floors, including both soil and concrete floors, tested positive for cefotaxime resistant *E. coli*. Our estimate is higher than the 48 % prevelence of ESBL-producing *E. coli* detected in household and hospital settings in Jordan but lower than the 91 % prevalence reported in a poulty farm environment in the Netherlands [27,28]. Since *E. coli* is an indicator of fecal contamination, high *E. coli* prevalence on household floors suggests poor hygiene and sanitation, raising concerns about exposure by household members, especially young children [29]. Moreover, cefotaxime resistant *E. coli* on floors raises further concerns considering the possibility of horizontally transferring resistance genes to surrounding pathogenic microbial populations [30]. Therefore, surveillance of environmental ESBL *E. coli* is essential to track dissemination through environmental sources, including soils and household floors.

The presence of antibiotic resistance in *E. coli* has been mainly correlated to the occurrence of different β-lactamase genes, including mainly the ESBL genes along with some other plasmid-mediated resistance genes [31]. ESBL genes render the isolates resistant to all β-lactam class antibiotics and often to other antibiotic classes as well, causing difficult-to-treat infections that require the application of “last-resort” antibiotics such as carbapenems [[32], [33], [34]]. The majority (84.6 %) of the β-lactamase producers in this study had the *bla*_*CTX-M*_ gene, consistent with previous studies demonstrating the high prevalence of this gene among cefotaxime resistant isolates [[35], [36], [37]]. In fact, *bla*_*CTX-M*_ is one of the foremost groups of ESBL genes among environmental sources and nosocomial infections, which might be because of the presence of multiple clones of the CTX-M enzyme resulting from the epidemic pattern of dissemination of several mobile genetic elements [35,38]. The high presence of the bla_*CTX-M*_ gene on household floors, consistent with previous evidence of detection in farming soil [39,40], requires attention and reinstates the necessity to find an efficient solution to contain the spread of the gene among other pathogenic organisms. The second most prominent β-lactamase gene in our study was *bla*_*TEM*_ (22.0 %). TEM has been reported to be a common β-lactamase gene in gram-negative organisms, specifically *E. coli*, and accounts for ampicillin resistance in the Enterobacteriaceae group [41,42]. Since 97.8 % of the isolates in our study were resistant to ampicillin, this might partially be attributed to the presence of bla_*TEM*_ gene. Another 6.6 % of the isolates were positive for *bla*_*SHV*_ and none of the isolates were found to be positive for *bla*_*OXA*_, which is consistent with previous studies showing a lower prevalence of these genes among cefotaxime resistant isolates [37,43,44].

The persistence of MDR *E. coli* in soil samples has been a major concern in developing countries [12]. The extensive use of antibiotics in vegetable farming and animal production leads to the dissemination of antibiotics in the environment, exacerbating the persistence of resistance among pathogens [[45], [46], [47]]. Two recent studies conducted in Bangladesh and Tanzania have isolated 12.6 % and 10 % MDR *E. coli* from soil samples collected from household front yards, suggesting soils in residential areas can harbor MDR organisms due to poor sewage disposal or unsafe management of manure [12,48]. A concerning finding is that all isolates in our study were MDR *E. coli*, and one isolate was XDR as defined by previous studies [[20], [21], [22], [23], [24]]. Exposure to MDR pathogenic strains on floors can lead to difficult-to-treat infections among members of these households, especially children [29]. Moreover, *E. coli* has been reported to be easily transmitted through aerosol formation [49], which raises further concerns regarding the dissemination of these resistant organisms into the environment and contributing to the rise in resistance among surrounding microbial communities through exerting selective pressure.

Diarrheagenic *E. coli* (DEC) is the primary cause of foodborne infection among all age groups and demographics, with symptoms not only limited to diarrhea but also gastroenteritis, nutrient malabsorption and inflammation [50]. In our study, 8/91 (8.8 %) of the isolates were identified as diarrheagenic strains, raising health concerns for household members, considering the samples were collected from floors within the interior of the households. The virulence genes of these DEC may confer additional benefits for the organism to survive under environmental stress as well as provide an evolutionary advantage compared to other microbial populations in the same environment [51]. Besides, all 8 DEC isolates were resistant to ≥5 of the antibiotics tested, therefore classified as MDR, raising concern regarding the treatment of the infections caused by these isolates using our current clinical arrangements. Although there are studies suggesting that the virulence of the DEC strains does not directly influence the persistence or survival of these strains in the environment [52], both climatic and anthropogenic factors in different ecological niches have to be considered for understanding the actual risk exacerbated by the MDR DEC strains present on household floors.

ExPEC are hypothesized to be opportunistic pathogens. They occupy a niche in human and animal intestines, serving as reservoirs of virulence genes, but are only capable of colonizing the extraintestinal sites of the host, making them the leading cause of adult bacteremia, the second leading cause of neonatal meningitis and the cause of the vast majority of urinary tract infections [8,53]. It is difficult to retrace the origin of ExPEC causing human infections but it is well established that the transmission occurs through various routes including contaminated food or direct contact with domestic animals [53]. Domestic animals were common among households in our study, which may explain the high prevalence (84.6 %) of isolates with at least one ExPEC virulence gene in our study, even though only 5.5 % of isolates were definitively classified as ExPEC due to being positive for at least three virulence genes. Notably, all of the ExPEC strains were resistant to ≥6 of the 15 antibiotics tested. The indiscriminate use of antibiotics in agriculture and veterinary medicine may generate selection pressure for the environmental ExPEC strains and make the treatment of such infections increasingly challenging [54]. It is important to investigate the ecological behavior and antibiotic resistance patterns of ExPEC strains to tackle future economic and public health concerns.

Biofilms are considered hotspots for horizontal gene transfer, with a high potential to transfer resistance genes [55,56]. Soil biofilms are of primary concern since soil is considered a reservoir for different antibiotic resistance genes and is a fundamental part of human or animal lives, establishing a medium for the transmission of resistant pathogens to humans or animals [57]. *E. coli* is a well-accepted model organism for *in vivo* studies about biofilm formation on abiotic surfaces because many cell surface components including flagella, type I fimbriae, colonic acid, poly(β-1,6-GlcNAc)], autotransporters and other outer membrane proteins were found in *E. coli* K-12 strain [[58], [59], [60]]. Besides, *E. coli* biofilms are the major causative agents for medical-device associated infectivity. For example, UPEC strains have been frequently associated with biofilms formed in the lumen of catheters, where they resist antibiotics and shear stress [61]. Biofilms also assist *E. coli* in mounting resistance against antibiotics, mostly accredited to putative multidrug resistant pumps, reduced antimicrobial diffusion, and reduced growth rates [[62], [63], [64]]. The extent of biofilm formation is influenced by different physicochemical parameters, as well as by the surrounding microbial communities [65]. The biofilm forming potential of environmental *E. coli* isolates and factors that contribute to this potential have not been well investigated [66]. In our study, we found that the biofilm forming capacity of cefotaxime-resistant *E. coli* was higher at 25 °C compared to 37 °C, which is consistent with previous studies [67]. Considering the historical average temperature of Bangladesh being 25.75 °C [68], the majority of the isolates will be capable of inducing biofilm formation within the household indoor environment. Notably, at 25 °C, 4 out of 5 ExPEC isolates and 4 out of 8 DEC isolates showed strong biofilm forming tendency, which is concerning considering the resistance or virulence spreading potential of biofilms.

There are some limitations of this study. Firstly, we conducted PCR for four major ESBL genes, so there might be a chance of underdetection of genes. Secondly, the study findings may not be generalized to other environmental conditions because the survival and distribution of microbes in a tropical environment are significantly different than other environmental conditions. In addition, we did not investigate the isolates for their plasmids. A large amount of antimicrobial resistance in the environment often correlates with resistance-trait plasmids [69], hence plasmid-mediated resistance was left undetected. Finally, our sample size was small and therefore we may have missed associations. However, this study provides insight into the resistance and pathogenesis of *E. coli* isolates from household floors in a rural low-income country setting and can form a foundation for future studies on AMR transmission via floors.

## 4 Conclusion

There is a rising concern worldwide regarding the contribution of environmental compartments including hands, surfaces, and soil in transmitting enteric bacteria and antimicrobial resistant bacteria. Our study demonstrates the widespread presence of cefotaxime resistant *E. coli* on household floors in a rural setting. A considerable percentage of the resistant isolates was diarrheagenic and ExPEC, as determined by molecular confirmation. Notably, all cefotaxime resistant *E. coli* isolates, including the pathogenic isolates, were MDR. Coexistence of virulence and resistance against multiple classes of antibiotics poses a severe threat of “difficult-to-treat” infections. In rural Bangladesh and similar settings, these risks are exacerbated by limited access to healthcare facilities. Future studies should quantify AMR *E. coli* in different environmental compartments, including household floors, to indicate the extent of fecal contamination and antimicrobial resistance in such settings. Studies focusing on elucidating the fate as well as the origin of these pathogenic organisms are crucial to design preventive steps for controlling exposure and transmission. All these findings will necessitate preventive measures and facilitate interventions for improved sanitation and hygiene practices to improve health among vulnerable populations.

## 5 Materials and methods

### 5.1 Swab collection

Floor swab samples were collected from 49 households in villages in Sirajganj district in northwestern Bangladesh, with ethical consent of the Institutional Review Board (IRB) of the International Centre for Diarrhoeal Disease Research, Bangladesh (icddr,b). The range of location coordinates of the sampling households are from latitude (24.42133–24.54405) to longitude (89.42954–89.36405) and from latitude (24.47101–24.45911) to longitude (89.28701–89.43716). Households were enrolled if they had a child <2 years old, had either a soil or concrete floor and did not have a recent case of anthrax. Field staff collected one floor swab sample in each household using a sterile Whirl-Pak® PolySponge™ bag pre-hydrated with 10 ml of HiCap™ Neutralizing Broth (Nasco, USA). A sterilized 50 cm × 50 cm stencil was laid next to the head of the bed where the child usually sleeps to mark the floor sampling area, avoiding areas under furniture, mats or carpets. Field staff swabbed the area within the stencil once horizontally and once vertically. Samples were preserved on ice and transported ensuring a temperature between 4 °C and 10 °C to the Laboratory of Environmental Health (LEH), icddr,b, Dhaka [70].

### 5.2 Swab processing and isolating cefotaxime resistant *E. coli*

The samples were preserved overnight at 4 °C and processed within 24 h from sampling time. Samples were brought to room temperature before processing. Using a sterile graduated cylinder, 100 ml autoclaved deionized water was added into the Whirl-Pak® Hydrated PolySponge™ Bag containing the sponge. The sponge was massaged from outside for 15 s and then gently swirled for 15 s, followed by decanting the liquid into a sterile Whirl-Pak bag [71]. The process was repeated a total of three times to generate a 300 ml solution. Using a sterile disposable pipette, 10 ml solution was immediately removed from the bottom of the elution bag and added into a fresh Whirlpak containing 90 ml autoclaved deionized water to create a 100 mL final aliquot with 10-fold dilution. This step was repeated to create a second 100 mL aliquot with 100-fold dilution. Colilert-18 media was added to each 100 mL aliquot, and 80 μL of filter-sterilized cefotaxime solution (5 mg/mL) was added after Colilert-18 media was completely dissolved [[72], [73], [74]]. Samples were transferred into Quanti/Tray 2000 and incubated at 35 °C for 22 h [75]. Following incubation, the Quanti/Tray cells with presumptive cefotaxime resistant *E. coli* were identified as those fluorescing under UV light. The most probable number (MPN) of cefotaxime resistant *E. coli* was quantified as per the MPN table [76] and reported as MPN/100 ml of the eluted floor swab samples. Up to three fluorescent cells were chosen in random order from individual Quanti/Trays and 100 μL of sample was collected from selected cells after sterilizing the back of the tray using 70 % ethanol. The presumptive *E. coli* samples from the positive Quanti/Tray cells were streaked on non-selective MacConkey Agar (BD, USA) plates and incubated at 37 °C for 24 h. Colonies appearing pink were confirmed as *E. coli*. From each plate, one representative isolate was chosen for further analysis, yielding a total of 91 isolates. Each isolate represented one positive Quanti/Tray cell from the eluted swabs. *E. coli* strain ATCC 25922 was used as a positive control for MacConkey agar. Field blanks, lab blanks, field duplicates and lab replicates were processed for QA/QC.

### 5.3 Detection of antimicrobial resistance genes

Confirmed cefotaxime-resistant *E. coli* colonies from MacConkey Agar plates were incubated overnight in 3 ml Luria-Bertani (LB) broth for enrichment. Following the boiling lysis method, DNA was extracted from the enriched broth [77]. An extraction blank was generated for each batch of DNA extraction. PCR analysis was performed to detect ESBL genes *bla*_SHV_*, bla*_OXA_*, bla*_TEM_ and *bla*_CTX-M_ as per a previously established protocol [67]. Primer sequences for all target genes in the study with their subsequent product sizes are provided in Supplementary Table S2. Positive controls were used from previously conducted studies [77], a no-template control was used as a negative control for PCR, and the extraction blanks were processed along with samples. To detect successful PCR amplification and the presence of the targeted gene, post-PCR amplification confirmation was conducted using agarose gel electrophoresis. The gel was prepared using 1 % agarose with freshly prepared Tris-Borate EDTA (0.5X TBE) buffer. The gels were examined for the presence of bands in desired band size after viewing through the GelDoc Go Imaging System (BIORAD, California, USA) and images were saved for further analysis. The same procedures for QA/QC and post-PCR amplification were executed for all the PCR reactions described below.

### 5.4 Detecting diarrheagenic *E. coli* (DEC) genes

DNA from the cefotaxime resistant *E. coli* isolates was analyzed by PCR to detect diarrheagenic genes, as per a previously established protocol [77]. A multiplex PCR specific for eight genes was conducted at first targeting ETEC (*estA, eltB)*, EPEC (*bfpA, eae*), EAEC (*aaiC, aat)*, and EIEC (*iaa, ipaH*) detection, the genes corresponding to most prevalent *E. coli* pathotypes. Another simplex PCR was conducted for the *stx* gene for the detection of EHEC (*stx1, stx2)*. The primer sequences for the corresponding genes and their subsequent product sizes are provided in Supplementary Table S2.

### 5.5 Detecting ExPEC genes

DNA from the cefotaxime resistant *E. coli* isolates was further screened for detection of seven extraintestinal pathogenicity (ExPEC) associated genes, including *focG, kpsMII, papA, sfaS, afa, hlyD* and *iutA*, by multiplex PCR as per an established protocol [77]. The primer sequences for the corresponding genes and their subsequent product sizes are provided in Supplementary Table S2.

### 5.6 Determination of antibiotic resistance profile

The antimicrobial sensitivity profiling of cefotaxime resistant isolates was characterized following the Clinical Laboratory Standards Institute (CLSI) guidelines [78]. The susceptibility profiles of all the isolates against 15 antibiotics, ampicillin (AMP, 10 μg), cefuroxime (CXM, 30 μg), cefotaxime (CTX, 30 μg), cefepime (FEP, 30 μg), aztreonam (ATM, 30 μg), meropenem (MEM, 10 μg), gentamicin (CN, 10 μg), nitrofurantoin (F, 300 μg), tetracycline (TE, 30 μg), tigecycline (TG, 15 μg), ciprofloxacin (CIP, 5 μg), sulfamethoxazole-trimethoprim (SXT, 25 μg), chloramphenicol (C, 30 μg), piperacillin-tazobactam (TPZ, 110 μg) and fosfomycin (FOS, 200 μg), were established following the Kirby-Bauer disc diffusion assay using commercially utilized disks from Thermo Fisher Scientific™ (Waltham, USA). *E. coli* ATCC 25922 strain was used as a positive control and the procedure was replicated three times. The average diameter of the zone of inhibition (mm) was used to classify isolates as sensitive, intermediate, and resistant as per the CLSI and EUCAST guidelines [78,79].

### 5.7 Biofilm forming assay

The biofilm forming ability of the cefotaxime resistant *E. coli* isolates was examined by conducting the quantitative adherence assay at both 25 °C and 37 °C [80]. The results of the biofilm containing microtiter plates (OD) were detected at 590 nm wavelength using an ELISA plate reader (BioTek, Vermont, USA). The isolates were classified as strong biofilm former, moderate biofilm former, weak biofilm former or non-biofilm former [37].

### 5.8 Statistical analysis

Microsoft Excel 2019 and R (version 4.3.2) were used for statistical analysis. The presence of resistance and virulence genes was tabulated as a binary variable, 1 depicting the presence of the tested genes and 0 depicting the absence of the tested genes for each set of analyses. For the correlation plot, antibiogram results were also interpreted using the binary format, 1 depicting resistant or intermediate and 0 depicting sensitive isolates. Correlations between the detection of resistance genes, DEC genes, ExPEC genes, phenotypic resistance and biofilm forming tendency were assessed using the ‘cor’ function, and the Fisher exact test was utilized to determine significant (p < 0.05) correlations. In order to visualize correlations between these variables for interpretation, the ‘corrplot’ function was used [81]. The correlation plot excluded those variables to which isolates demonstrated 100 % resistance or sensitivity.

## Funding

This study was supported by 10.13039/100000002National Institute of Health (NIH), grant number R01 HD 108196.

## Data availability statement

Data associated with this study have not been deposited into a publicly available repository. Data will be made available on request.

## CRediT authorship contribution statement

**Tahani Tabassum:** Writing – review & editing, Writing – original draft, Visualization, Methodology, Data curation. **Md. Sakib Hossain:** Writing – review & editing, Visualization, Validation, Methodology, Investigation, Data curation. **Ayse Ercumen:** Writing – review & editing, Validation, Project administration, Investigation, Conceptualization. **Jade Benjamin-Chung:** Writing – review & editing, Validation, Project administration, Investigation, Funding acquisition. **Md. Foysal Abedin:** Software, Formal analysis, Data curation. **Mahbubur Rahman:** Writing – review & editing, Project administration, Funding acquisition. **Farjana Jahan:** Writing – review & editing, Project administration, Funding acquisition. **Munima Haque:** Writing – review & editing, Supervision. **Zahid Hayat Mahmud:** Writing – review & editing, Validation, Supervision, Resources, Project administration, Investigation, Funding acquisition, Conceptualization.

## Declaration of competing interest

The authors declare that they have no known competing financial interests or personal relationships that could have appeared to influence the work reported in this paper.
