# Supplementary material for: Isolation and characterization of cefotaxime resistant Escherichia coli from household floors in rural Bangladesh
Source: Heliyon. 2024 Jul 9;10(14):e34367. doi: 10.1016/j.heliyon.2024.e34367 (PMC11305256; doi:10.1016/j.heliyon.2024.e34367)
Supplement: Multimedia component 1 [file mmc1.docx]

Supplementary Table S1: Estimated number of cefotaxime-resistant *E. coli* in the soil swabs.

| **Numerical ID** | **LARGE FLUOR WELLS** | **SMALL FLUOR WELLS** | **MPN/100 ml** |
| --- | --- | --- | --- |
| 20019 | 1 | 0 | 100.0 |
| 20008 | 1 | 0 | 10.0 |
| 20021 | 1 | 0 | 100.0 |
| 20004 | 26 | 4 | 399.0 |
| 20018 | 7 | 3 | 96.0 |
| 20025 | 49 | 45 | 17329.0 |
| 20034 | 33 | 2 | 548.0 |
| 20002 | 4 | 1 | 52.0 |
| 20012 | 12 | 1 | 146.0 |
| 20014 | 49 | 48 | >24196.0 |
| 20023 | 19 | 3 | 272.0 |
| 20050 | 48 | 22 | 2987.0 |
| 20013 | 46 | 10 | 1467.0 |
| 20016 | 26 | 6 | 443.0 |
| 20031 | 48 | 44 | 8297.0 |
| 20055 | 49 | 48 | >24196.0 |
| 20059 | 22 | 2 | 309.0 |
| 20056 | 45 | 48 | 2075.0 |
| 20030 | 1 | 0 | 10.0 |
| 20051 | 23 | 4 | 355.0 |
| 20028 | 42 | 4 | 932.0 |
| 20043 | 49 | 17 | 2909.0 |
| 20044 | 29 | 4 | 480.0 |
| 20047 | 9 | 5 | 153.0 |
| 20048 | 14 | 0 | 161.0 |
| 20052 | 49 | 48 | >24196.0 |
| 20011 | 42 | 6 | 988.0 |
| 20020 | 49 | 40 | 11199.0 |
| 20026 | 4 | 0 | 41.0 |
| 20039 | 22 | 2 | 309.0 |
| 20045 | 5 | 0 | 52.0 |
| 20049 | 1 | 0 | 10.0 |
| 20042 | 1 | 0 | 10.0 |
| 20054 | 5 | 0 | 52.0 |
| 20065 | 1 | 0 | 10.0 |
