# Supplementary material for: Isolation and characterization of cefotaxime resistant Escherichia coli from household floors in rural Bangladesh
Source: Heliyon. 2024 Jul 9;10(14):e34367. doi: 10.1016/j.heliyon.2024.e34367 (PMC11305256; doi:10.1016/j.heliyon.2024.e34367)
Supplement: Multimedia component 2 [file mmc2.docx]

Supplementary Table S2: Primer sequences and subsequent product size for PCR detection of ESBL genes, DEC genes and ExPEC genes.

|  | **Target**  **Gene** | **Primer**  **name** | **Sequence**  **(5’ → 3’)** | **Product**  **size (bp)** | **Reference** |
| --- | --- | --- | --- | --- | --- |
| **ESBL primers** | *bla_SHV_* | SHV-F | CTTTATCGGCCCTCACTCAA | 237 | [1] |
|  |  | SHV-R | AGGTGCTCATCATGGGAAAG |  |  |
|  | *bla_TEM_* | TEM-F | CGCCGCATACACTATTCTCAGAATGA | 445 |  |
|  |  | TEM-R | ACGCTCACCGGCTCCAGATTTAT |  |  |
|  | *bla_CTX-M_* | CTX-M-F | ATGTGCAGYACCAGTAARGTKATGGC | 593 |  |
|  |  | CTX-M-R | TGGGTRAARTARGTSACCAGAAYCAGCGG |  |  |
|  | *bla_OXA_* | OXA-F | ACACAATACATATCAACTTCGC | 813 |  |
|  |  | OXA-R | AGTGTGTTTAGAATGGTGATC |  |  |
| Intestinal pathogenic primers | *estA* | **ST-F** | *GCTAAACCAGTAGAGGTCTTCAAAA* | 147 | [2] |
|  |  | **ST-R** | *CCCGGTACAGAGCAGGATTACAACA* |  |  |
|  | *eltB* | **LT-F** | *CACACGGAGCTCCTCAGT C* | 508 | [3] |
|  |  | **LT-R** | *CCCCCAGCCTAGCTTAGTTT* |  |  |
|  | *bfpA* | **bfpA-F** | *GGAAGTCAAATTCATGGGGG* | 300 | [3] |
|  |  | **bfpA-R** | *GGAATCAGACGCAGACTGGT* |  |  |
|  | *Eae* | **eae-F** | *CCCGAATTCGGCACAAGCATAAGC* | 881 | [4] |
|  |  | **eae-R** | *CCCGGATCCGTCTCGCCAGTATTCG* |  |  |
|  | *aaiC* | **aaiC-F** | *ATTGTCCTCAGGCATTTCAC* | 215 | [3] |
|  |  | **aaiC-R** | *ACGACACCCCTGATAAACAA* |  |  |
|  | *Aat* | **pcvd432-F** | *CTGGCGAAAGACTGTATCAT* | 650 | [5] |
|  |  | **pcvd432-R** | *CAATGTATAGAAATCCGCTGTT* |  |  |
|  | *Iaa* | **ial upper** | *CTGGATGGTATGGTGAGG* | 320 | [6] |
|  |  | **ial lower** | *GGAGGCCAACAATTATTTCC* |  |  |
|  | *ipaH* | **Shig-1** | *TGGAAAAACTCAGTGCCTCT* | 424 | [7] |
|  |  | **Shig-2** | *CCAGTCCGTAAATTCATTCT* |  |  |
|  | *stx1* | **stx1F** | *CACAATCAGGCGTCGCCAGCGCACTTGCT* | 606 | [8] |
|  |  | **stx1R** | *TGTTGCAGGGATCAGTGGTACGGGGATGC* |  |  |
|  | *stx2* | **stx2F** | *CCACATCGGTGTCTGTTATTAACCACACC* | 372 | [9] |
|  |  | **stx2R** | *GCAGAACTGCTCTGGATGCATCTCTGGTC* |  |  |
| **ExPEC primers** | *focG* | focG_106F | CGTACCTGTACCATTGGTAATGGAGG | 366 | [10] |
|  |  | focG_471R | TGAATTAATACTTCCCGCACCAGC |  |  |
|  | *kpsMII* | kpsMII_121F | GCGCATTTGCTGATACTGTTG | 452 |  |
|  |  | kpsMII_572 | GGGAACATGATGCAGGAGATG |  |  |
|  | *papA* | papA_67F | ATGGCAGTGGTGTCTTTTGGTG | 717 |  |
|  |  | papA_+202R | CGTCCCACCATACGTGCTCTTC |  |  |
|  | *sfaS* | sfaS_210F | GTCTCTCACCGGATGCCAGAATAT | 138 |  |
|  |  | sfaS_347R | GCATTACTTCCATCCCTGTCCTG |  |  |
|  | *Afa* | afa F | GGCAGAGGGCCGGCAACAGGC | 594 |  |
|  |  | afa R | CCCGTAACGCGCCAGCATCTC |  |  |
|  | *hlyD* | hlyD_92F | CTCCGGTACGTGAAAAGGAC | 904 |  |
|  |  | hlyD_995R | GCCCTGATTACTGAAGCCTG |  |  |
|  | *iutA* | iutA_674F | ATCGGCTGGACATCATGGGAAC | 314 |  |
|  |  | iutA_987R | CGCATTTACCGTCGGGAACGG |  |  |

**References**

[1] H. Fang, F. Ataker, G. Hedin, K. Dornbusch, Molecular epidemiology of extended-spectrum β-lactamases among Escherichia coli isolates collected in a Swedish hospital and its associated health care facilities from 2001 to 2006, J. Clin. Microbiol. 46 (2008) 707–712. https://doi.org/10.1128/JCM.01943-07.

[2] T.V. Nguyen, P. Le Van, C. Le Huy, K.N. Gia, A. Weintraub, Detection and characterization of diarrheagenic Escherichia coli from young children in Hanoi, Vietnam, J. Clin. Microbiol. 43 (2005) 755–760.

[3] P.K. Talukdar, M. Rahman, M. Rahman, A. Nabi, Z. Islam, M.M. Hoque, H.P. Endtz, M.A. Islam, Antimicrobial resistance, virulence factors and genetic diversity of Escherichia coli isolates from household water supply in Dhaka, Bangladesh, PLoS One. 8 (2013) e61090.

[4] E. Oswald, H. Schmidt, S. Morabito, H. Karch, O. Marches, A. Caprioli, Typing of intimin genes in human and animal enterohemorrhagic and enteropathogenic Escherichia coli: characterization of a new intimin variant, Infect. Immun. 68 (2000) 64–71.

[5] J.A. Mohammed, H.L. DuPont, Z.-D. Jiang, J. Flores, L.G. Carlin, J. Belkind-Gerson, F.G. Martinez-Sandoval, D. Guo, A.C. White Jr, P.C. Okhuysen, A single-nucleotide polymorphism in the gene encoding osteoprotegerin, an anti-inflammatory protein produced in response to infection with diarrheagenic Escherichia coli, is associated with an increased risk of nonsecretory bacterial diarrhea in North Ame, J. Infect. Dis. 199 (2009) 477–485.

[6] G. Frankel, J.A. Giron, J. Valmassoi, G.K. Schoolnik, Multi‐gene amplification: simultaneous detection of three virulence genes in diarrhoeal stool, Mol. Microbiol. 3 (1989) 1729–1734.

[7] D. Lüscher, M. Altwegg, Detection of shigellae, enteroinvasive and enterotoxigenic Escherichia coli using the polymerase chain reaction (PCR) in patients returning from tropical countries, Mol. Cell. Probes. 8 (1994) 285–290.

[8] M.A. Islam, A.E. Heuvelink, E. De Boer, P.D. Sturm, R.R. Beumer, M.H. Zwietering, A.S.G. Faruque, R. Haque, D.A. Sack, K.A. Talukder, Shiga toxin-producing Escherichia coli isolated from patients with diarrhoea in Bangladesh, J. Med. Microbiol. 56 (2007) 380–385.

[9] A.E. Heuvelink, N. Van de Kar, J. Meis, L.A.H. Monnens, W.J.G. Melchers, Characterization of verocytotoxin-producing Escherichia coli O157 isolates from patients with haemolytic uraemic syndrome in Western Europe, Epidemiol. Infect. 115 (1995) 1–3.

[10] M.S. Hossain, S. Ali, M. Hossain, S.Z. Uddin, M. Moniruzzaman, M.R. Islam, A.M. Shohael, M.S. Islam, T.H. Ananya, M.M. Rahman, M.A. Rahman, M. Worth, D. Mondal, Z.H. Mahmud, ESBL Producing Escherichia coli in Faecal Sludge Treatment Plants: An Invisible Threat to Public Health in Rohingya Camps, Cox’s Bazar, Bangladesh, Front. Public Heal. 9 (2021). https://doi.org/10.3389/fpubh.2021.783019.
